# Transcriptomic insights into the epigenetic modulation of turnip mosaic virus evolution in *Arabidopsis thaliana*

**DOI:** 10.1186/s12864-024-10798-x

**Published:** 2024-09-30

**Authors:** María J. Olmo-Uceda, Silvia Ambrós, Régis L. Corrêa, Santiago F. Elena

**Affiliations:** 1 Instituto de Biología Integrativa de Sistemas (I 2 SysBio), CSIC-Universitat de València, Catedrático Agustín Escardino 9, Paterna, Valencia, 46980 Spain; 2 Departmento de Genética, Universidade Federal do Rio de Janeiro (UFRJ), Rio de Janeiro, Brazil; 3 Santa Fe Institute, 1399 Hyde Park Road, Santa Fe, NM 87501 USA

**Keywords:** Adaptation, Epigenetics, Experimental evolution, Gene expression, *Potyvirus rapae*, Systems biology, Virus evolution, Virus-host interactions

## Abstract

**Background:**

Plant-virus interaction models propose that a virus’s ability to infect a host genotype depends on the compatibility between virulence and resistance genes. Recently, we conducted an evolution experiment in which lineages of turnip mosaic virus (TuMV) were passaged in *Arabidopsis thaliana* genotypes carrying mutations in components of the DNA methylation and the histone demethylation epigenetic pathways. All evolved lineages increased infectivity, virulence and viral load in a host genotype-dependent manner.

**Results:**

To better understand the underlying reasons for these evolved relationships, we delved into the transcriptomic responses of mutant and WT plant genotypes in mock conditions and infected with either the ancestral or evolved viruses. Such a comparison allowed us to classify every gene into nine basic expression profiles. Regarding the targets of viral adaptation, our analyses allowed the identification of common viral targets as well as host genotype-specific genes and categories of biological processes. As expected, immune response-related genes were found to be altered upon infection. However, we also noticed the pervasive over-representation of other functional groups, suggesting that viral adaptation was not solely driven by the level of expression of plant resistance genes. In addition, a significant association between the presence of transposable elements within or upstream the differentially expressed genes was observed. Finally, integration of transcriptomic data into a virus-host protein-protein interaction network highlighted the most impactful interactions.

**Conclusions:**

These findings shed extra light on the complex dynamics between plants and viruses, indicating that viral infectivity depends on various factors beyond just the plant’s resistance genes.

**Supplementary Information:**

The online version contains supplementary material available at 10.1186/s12864-024-10798-x.

## Introduction

Plants and viruses engage in complex interactions that trigger defense and counter-defense mechanisms, often leading to coevolution and reciprocal adaptation. Plant immune processes play a crucial role in antiviral defense [1, 2]. These antiviral factors fall into two categories: basal, which are preexisting and limit viral spread within and between cells, and inducible, which activate upon infection and prevent systemic movement and replication. Inducible mechanisms involve genes that induce broad-scale changes in plant physiology through various signaling pathways, while basal mechanisms involve cell protein alleles whose interaction with viral factors is altered [3]. These changes include local cell death [4], upregulation of nonspecific pathogen responses throughout the plant (systemic acquired resistance -SAR- and induced systemic resistance -ISR-) [4, 5], and activation of RNA-silencing-based resistance, involved in both basal and inducible mechanisms [4, 6, 7].

RNA-based immunity starts by recognizing and degrading double-stranded RNAs (dsRNA). During viral replication, Dicer-like (DCL) proteins cut viral dsRNAs into small RNAs (sRNA), which are then loaded into Argonaute (AGO) proteins to silence complementary RNAs [7]. The *Arabidopsis thaliana* genome has four DCL genes, with most viral siRNAs depending on the DCL4 protein. However, strong antiviral defense may also require the hierarchical activity of DCL2 and DCL3 [8]. Enhancing the silencing response involves RNA-dependent RNA polymerases (RDR), which produce additional dsRNAs from the target [9].

These RNA-mediated defenses are part of a broader and evolutionarily conserved system that regulates gene expression and controls transposable elements (TE) through epigenetic modifications of DNA or histones [10]. DNA methylation occurs in all sequence contexts (CG, CHG, and CHH; with H different from G) and is initiated by RNA-directed DNA methylation (RdDM). This process uses sRNAs from the borders of TEs to limit their expression and affect neighboring gene expression [11]. RdDM involves several components, including DCL3, METHYLTRANSFERASE DOMAINS REARRANGED METHYLASE 2 (DRM2), RNA polymerase IV (POLIV), and RNA polymerase V (POLV) [12]. Non-canonical pathways also contribute to the RdDM pathway, such as DCL-independent mechanisms, POLII-derived mRNAs, or RDR6-derived dsRNAs [13]. Epigenetic marks are copied during replication by maintenance DNA methyltransferases like the plant-specific CHROMOMETHYLASE 3, and chromatin remodelers like DECREASED DNA METHYLATION 1 (DDM1) are needed to control gene expression and TE mobilization in sRNA-independent ways [14]. Histone modification is linked to DNA methylation marks. For instance, histone H3 trimethylation of lysine 9 (H3K9m3) is associated with TE repression, while H3K4m3 promotes gene expression. Both DNA methylation and histone modifications are reversible, with proteins like REPRESSOR OF SILENCING 1 (ROS1), INCREASE IN BONSAI METHYLATION 1 (IBM1), and JUMONJI14 (JMJ14) involved in demethylating DNA, H3K9, and H3K4, respectively [15–17]. Under stressful conditions or in epigenetically-deficient mutants, the chromatin environment around TEs and genes can change, affecting their expression [18].

The connection between the loss of DNA methylation factors and resistance or susceptibility to RNA virus infection has received limited attention. Research on turnip crinkle virus [19], two tobamoviruses [20], and turnip mosaic virus (TuMV) [21] has shown that *A. thaliana* mutants for various RdDM factors, chromatin remodelers, and histone modifiers exhibit varying susceptibility to infections based on the presence of repressive marks. Additionally, evolution experiments with mutants for innate immunity pathways and basal and inducible defense pathways have shown that viral populations rapidly adapt to the new selective pressures imposed by mutations in RdDM genes [22, 23] and histone modification genes [23], indicating these genes play a direct role in the infection cycle of RNA viruses.

All these studies require having an adequate virus-host experimental pathosystem. Members of the *Potyvirus* genus are the largest group of plant RNA viruses that infect a wide range of agronomic species and cause significant economic losses. Extensive studies have tackled the multifunctional roles of potyviral proteins and their interplay with host factors [24, 25]. TuMV is a prototypical member of the genus that infects a broad range of vegetable crops species. From a scientific point of view, TuMV has two particularly relevant properties: (*i*) its wide host range that includes both mono and dicotyledons and (*ii*) being the potyvirus with the highest incidence in wild populations of *A. thaliana* [26]. Therefore, the pathosystem composed by TuMV and *A. thaliana* has been the focus of extensive scientific studies, including the evolutionary ones led by our group.

A common observation in evolution experiments with potyviruses is that the transcriptomic response of *A. thaliana* changes as viral lineages adapt better to the new host [21, 27–29]. To investigate the role of epigenetic regulation of host genes involved in defense responses in the probability of an RNA virus establishing successful infections after spilling over from a reservoir to a naïve host, Ambrós et al. [23] evolved TuMV lineages in *A. thaliana* plants with mutations in DNA methylation and histone demethylation pathways. They found that evolved viral lineages became more infectious, virulent, and reached higher viral loads. Interestingly, the extent of these phenotypic changes depended on the mutated epigenetic pathway. For instance, *jmj14* mutants with affected histone methylation selected for more virulent viruses, while mutants unable to produce siRNAs (*dcl2 dcl3 dcl4*) and thus more susceptible to infection, selected for less virulent viral strains. In terms of genomic changes, all cistrons were shown to be under purifying selection, particularly strong upon the *NIb* cistron encoding the viral replicase. In contrast, the VPg protein was shown to be the most polymorphic one, with nine amino acid substitutions, all affecting a particular domain in the protein, being under positive selection (see Fig. 4 in [23]). All but one of these fixed mutations was host genotype-specific, rising independently in different hosts. Mutation R118H was the only one that found specifically associated to evolution in *ddm1* plants. We hypothesize that given the expected large-scale effects of mutations in epigenetic regulatory pathways on plant defenses and cellular homeostasis, virus evolution and adaptation to the new host will be affected. This impact can be direct, if epigenetic factors directly interact with virus factors, or indirect, if epigenetic regulation changes the expression of plant resistance genes that ultimately interact with viral factors or cause a significant physiological imbalance, creating a different cellular environment for the virus. To further understand the role of DNA methylation and histone modification pathways in TuMV adaptation, we examined differences in whole-genome transcriptomic responses to infection with the ancestral TuMV and derived viral lineages in their corresponding local host genotypes.

## Methods

### Plants, virus and experimental evolution

For full details of the evolution experiment, refer to [23]. Briefly, the study used wild-type A. *thaliana* accession Col-0 and four mutants: *dcl2 dcl3 dcl4*, *ddm1*, *polv* (all affecting RdDM), and *jmj14* (a histone demethylase). All plants were grown in a climate-controlled chamber with an 8-hour light period (LED lights at PAR 90–100 µmol m^−2^ s^−1^) at 24 °C, and a 16-hour dark period at 20 °C with 40% relative humidity. The soil mix was 50% DSM WNR1 R73454 substrate (Kekkilä Professional, Vantaa, Finland), 25% grade 3 vermiculite, and 25% 3–6 mm perlite. Pest management included introducing *Stratiolaelaps scimitus* and *Steinernema feltiae* (Koppert Co., Málaga, Spain).

TuMV infectious sap was prepared from TuMV-infected *Nicotiana benthamiana* plants inoculated with the plasmid p35STunos, containing a cDNA of the TuMV genome (GenBank AF30055.2) as described in [21]. This sequence corresponds to the YC5 strain from calla lily [30]. Symptomatic tissues were pooled, frozen with liquid nitrogen, and homogenized using a Mixer Mill MM400 (Retsch GmbH, Haan, Germany).

**Fig. 1:**
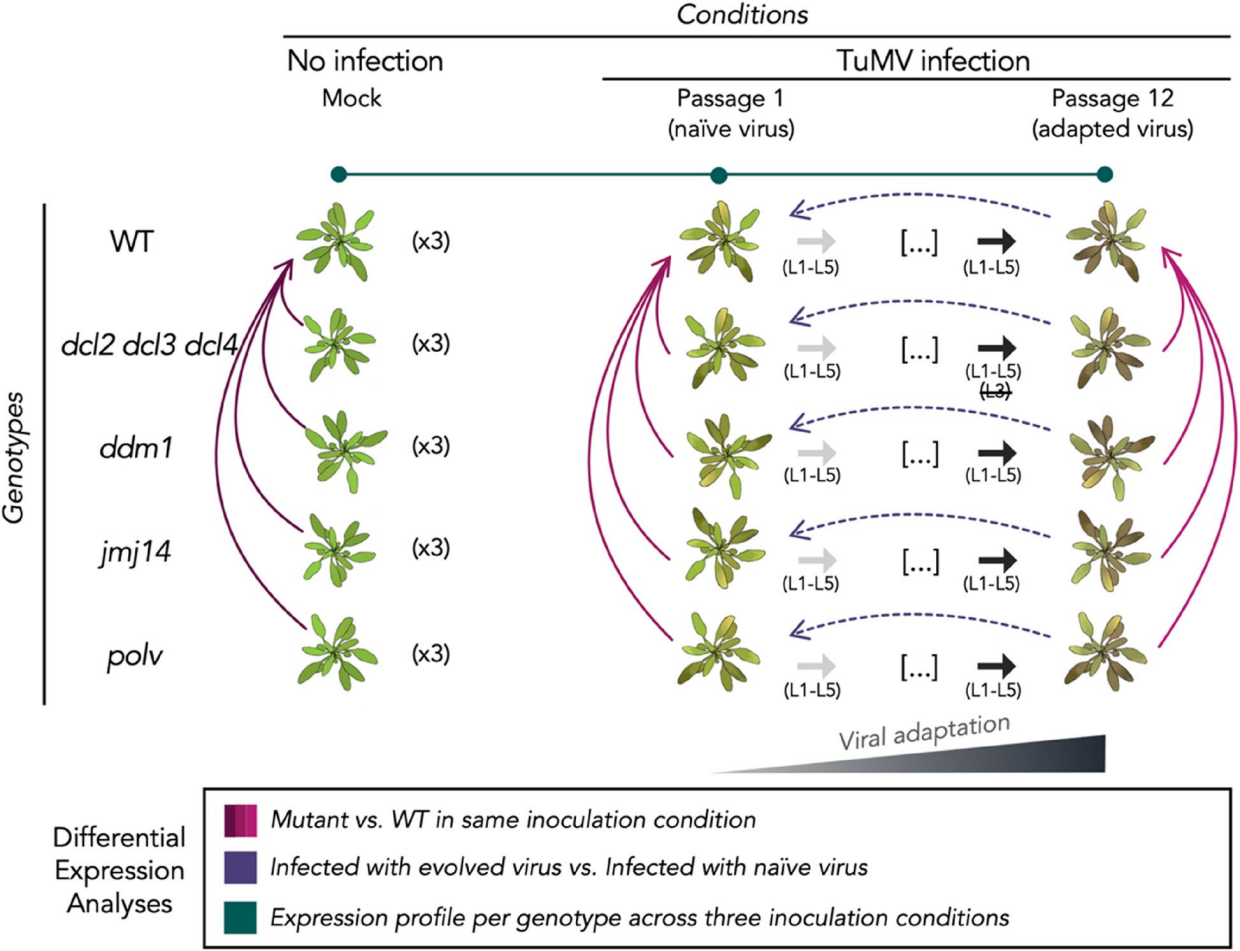
Schematic representation of the evolution experiment and the three different contrasts performed on the transcriptomic data. Considering three inoculation conditions: mock (no infection), infection with the ancestral virus (P1) and infection with the evolved viral lineages (P12), the five different plant genotypes and the five biological replicates (L1 – L5), three blocks of differential expression analyses were performed (represented by arrows, with the arrowhead pointing to the baseline of the contrast). Bourgogne: differences between each mutant and the WT in each condition. Purple: differences between the infection with adapted and the naïve virus for each genotype. The lineages were used as block variables. Green: the differential expression analyses between infection with naïve and mock and the differences between adapted and naïve infection (purple lines) led us to classify each gene expression in nine different profiles

Five TuMV lineages were evolved over twelve consecutive serial passages in each of the five genotypes (Fig. 1). To begin the experiment, ten *A. thaliana* plants per lineage and genotype were rubbed gently on two leaves with 5 µL of inoculum [0.1 g of stock diluted in 1 mL inoculation buffer (50 mM phosphate buffer pH 7.0, 3% PEG6000, 10% Carborundum)]. Plants were inoculated at growth stage 3.5 on the Boyes et al. scale to ensure phenological synchronization [31]. Subsequent passages were performed by harvesting symptomatic plants at 14 days post-inoculation (dpi), preparing infectious sap as described, and inoculating a new batch of ten healthy plants.

### Total RNA extractions and preparation of samples for HTS

Pools of ten infected symptomatic plants per lineage, genotype, and serial passage were frozen with liquid nitrogen and stored at − 80 ºC until homogenized into a fine powder. Aliquots of ~ 0.1 g were used for total RNA extractions using the Agilent Plant RNA Isolation Mini Kit (Agilent Technologies, Santa Clara, CA, USA). Three aliquots of total RNA per sample were separated and adjusted to 50 ng/µL.

For RNA-Seq, RNA from 70 to 90 mg of both infected and healthy plants was extracted using the GeneJET Plant RNA Purification Mini Kit (Thermo Fisher Scientific, Waltham, MA, USA) following the manufacturer’s instructions. RNA quality was checked with a NanoDrop One (Thermo Fisher Scientific) and agarose gel electrophoresis. RNA integrity and purity were verified using a Bioanalyzer 2100 (Agilent Technologies). Library preparation and Illumina sequencing were performed by Novogene Europe using a NovaSeq 6000 platform and a Lnc-stranded mRNA-Seq library method. This included ribosomal RNA depletion and directional library preparation, 150 paired-end sequencing, and 6 Gb raw data per sample. Novogene conducted quality checks on the libraries using a Qubit 4 Fluorometer (Thermo Fisher Scientific), qPCR for quantification, and a Bioanalyzer for size distribution detection.

### HTS data processing

The quality of the Fastq files was assessed using FASTQC (http://www.bioinformatics.babraham.ac.uk/projects/fastqc/) and MultiQC [32]. Paired reads were preprocessed with BBDuk (https://sourceforge.net/projects/bbmap/). During preprocessing, adapters were removed, the first ten 5’ nucleotides of each read were trimmed, and sequences from the 3’ end with an average quality below ten were cut off. Reads shorter than 80 nucleotides after processing were discarded. The parameters used were: *ktrim* = r, *k* = 31, *mink* = 11, *qtrim* = r, *trimq* = 10, *maq* = 5, *forcetrimleft* = 10, and *minlength* = 80.

The processed reads were then mapped to the TuMV isolate YC5 using the BWA-MEM algorithm [33]. The resulting SAM files were converted to binary and sorted with SAMtools [34]. Duplicates were marked using the MarkDuplicates method in GATK version 4.2.2.0 [35].

Next, the reads were mapped to the *A. thaliana* genome (TAIR10) using STAR version 2.7.9a [36] with the ‘alignReads’ mode and ‘GeneCounts’ quantification mode. These quantification results were used for transcriptome analysis. The resulting SAM files were again converted to binary and sorted with SAMtools [34].

All R and shell scripts created for this project are available at https://github.com/MJmaolu/evolution_TuMV_in_A.thaliana_Epigenetic_Mutants/tree/main.

### Host gene expression and functional annotation

The counts matrix was constructed from the STAR results (reversed-stranded column). Differential expression analyses were performed using DESeq2 v1.36.0 [37]. Genes were considered significant if they had an adjusted *P* < 0.05 (Benjamini and Hochberg method) and a |log_2_*FC*| > 1. This double criterion for defining differentially expressed genes (DEGs), the number of replicates per treatment (five) and our systems-wide description of the data (rather than focusing in particular genes) makes unnecessary further validation of expression levels by RT-qPCR [38, 39]. The first set of analyses compared the responses of each mutant genotype to the wild-type (WT) plants. This was done separately for mock-inoculated samples, samples inoculated with the ancestral virus, and samples inoculated with the evolved virus (bourgogne lines in Fig. 1). The second set of analyses compared samples infected with the evolved virus to those infected with the ancestral virus, treating samples infected by the same viral lineage as paired samples (purple lines in Fig. 1). The third set of analyses compared the differences between naïve infection and mock infection for each genotype (green lines in Fig. 1).

Functional enrichment analyses were performed using STRINGdb v2.8.4 [40] and clusterProfiler v4.4.4 [41].

All DEGs were screened for annotated transposable elements (TEs) within their open reading frames (ORFs) or up to one thousand nucleotides upstream of their promoters. This was done using the GenomicRanges package (v1.48.0) [42] and the *A. thaliana* transposon annotation file from TEtranscripts (https://labshare.cshl.edu/shares/mhammelllab/www-data/TEtranscripts/TE_GTF/, last accessed 11/09/2024). Functional enrichment analysis of the resulting genes was performed as previously described.

All processes were conducted using custom scripts in R version 4.2.1. These scripts, along with session information, are available in the mentioned github repository.

### Classification of gene expression across mock-ancestral viruses-evolved viruses into nine basic profiles

All the genes were classified in the nine possible profiles (Fig. 3 A) depending on the change in the expression between the three inoculation conditions. For evaluating the direction of changes (mock vs. ancestral and ancestral vs. evolved) the results obtained with DESeq2 for each genotype were used. For each of both changes, cases were assigned into three categories: (*i*) increasing expression if adjusted *P* < 0.05 and log_2_*FC* > 1, (*ii*) decreasing expression if adjusted *P* < 0.05 and log_2_*FC* < − 1 and (*iii*) non-affected otherwise.

### PPI network and viral protein enrichment

The *A. thaliana* – TuMV PPI published by Martínez et al. [43] was used. The DEGs selected in the contrast evolved vs. ancestral (purple lines in Fig. 1) were represented in the PPI. Node sizes indicate the number of genotypes where the gene is significantly altered (adjusted *P* < 0.05) with a |log_2_*FC*| > 1. The network was generated with Cytoscape version 3.10.0 [44]. We used the number of genes activated and repressed by the adapted viral lineages as a proxy of the probabilities for gene activation and repression in each host genotype. With these probabilities, we calculated the expected number of interactors altered for each viral protein (Fig. 7 C) and used a χ^2^ homogeneity test to evaluate whether some of the viral proteins were enriched or depleted in selected DEGs. Significant changes were considered when *P* < 0.05.

## Results and discussion

Figure 1 illustrates the experimental setup for the evolution study, highlighting specific contrasts in transcriptomic profiles. RNA-Seq analysis was performed on plants from four mutant lines and WT, infected with viral strains isolated at passages 1 (P1, the ancestral stage) and 12 (P12, the evolved lineages). Additionally, transcriptomes of mock-inoculated plants from these five genotypes were also analyzed. The *dcl2 dcl3 dcl4* mutant shows dysregulation of RNA silencing mechanisms, affecting antiviral siRNAs, RdDM, and endogenous siRNAs produced by RDR6-generated dsRNA. This mutant is notably susceptible to virus infection [8]. The *ddm1* and *polv* mutants exhibit altered DNA methylation mechanisms. DDM1 acts as a master regulator of transposable elements (TEs), and its deficiency leads to a loss of RdDM-independent DNA methylation and increased transposition events [45]. POLV plays a crucial role in RdDM by initiating CHH methylation on both DNA strands, specifically targeting short TEs and the edges of longer ones [12]. In *polv* mutants, there is a significant reduction in non-CG methylation across the genome, accompanied by disruption of various stress-related genes [46, 47]. The *jmj14* mutant shows elevated levels of H3K4 activation marks at its target sites [48].

### Summary of differentially expressed genes amongst infection treatments and plant genotypes

**Fig. 2:**
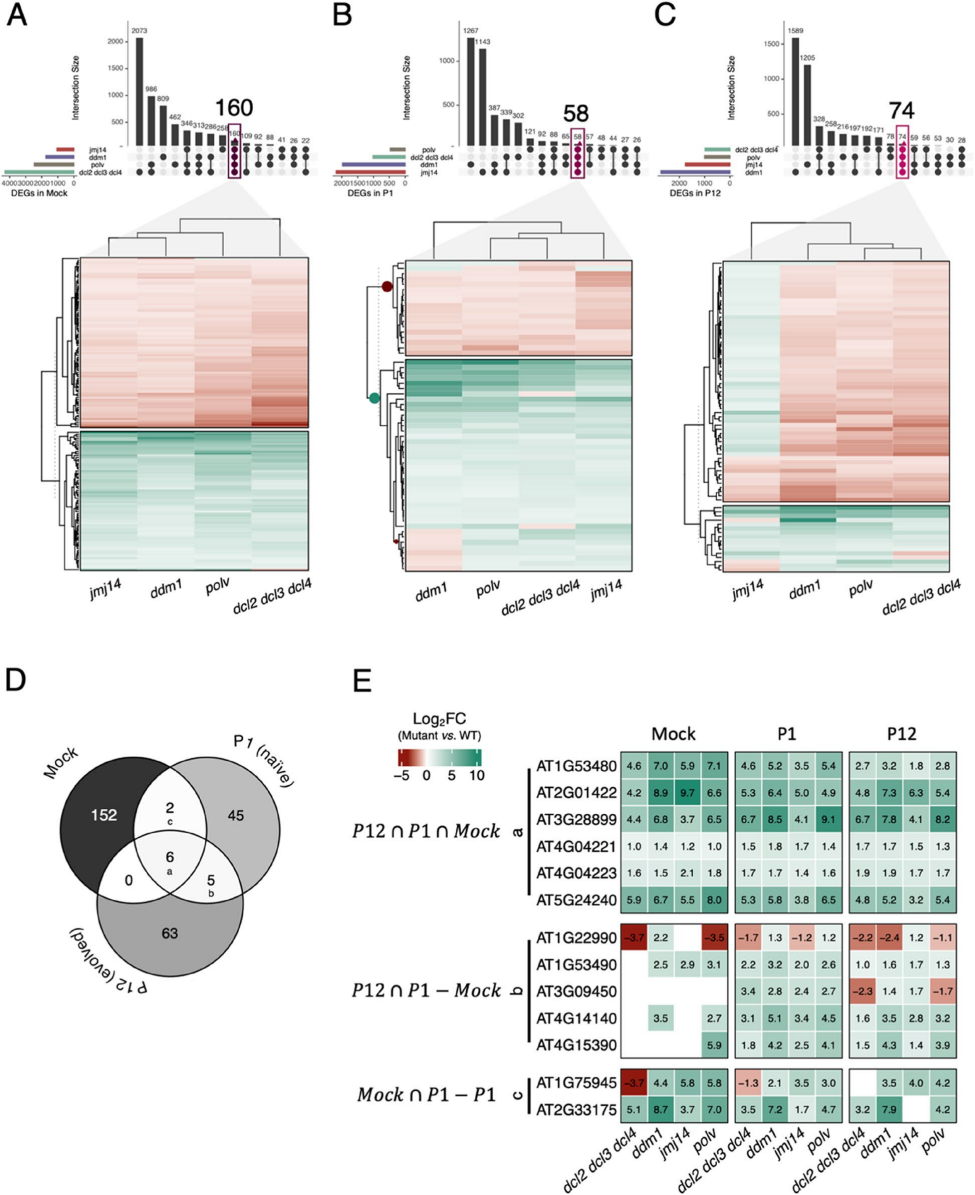
Distribution of differentially expressed genes (DEGs) compared to WT per plant genotype for mock-inoculated plants **A** plants inoculated with the ancestral virus **B** and plants inoculated with the evolved lineages. **C** Expression clustering for DEGs shared by all mutant plants are indicated in each case. **D** Distribution of DEGs shared by all the mutants (core genes) in mock-inoculated and plants infected with the ancestral (P1) and evolved (P12) viruses. **E** Gene expression (log_2_*FC* represent mutant vs. WT expression by inoculation condition) of the three overlapping sets of core genes in (D), only significant DEGs (adjusted *P* < 0.05) with |log_2_*F*C| > 1 expression levels are shown, rest in white

First, we analyzed the differences in the transcriptomes of the mutants with respect to the WT in each inoculation condition (Fig. 2 and Supplemental Fig. S1). These contrasts represent a first approach to discover differences in the way evolution has changed the interaction of evolved viruses with their host genotypes deficient in epigenetic components relative to their interaction with WT plants that are fully competent in all epigenetic regulation pathways. Figure 2 A shows the distribution of the number of differentially expressed genes (DEGs) for each of the four mutant plant genotypes compared to WT plants, non-inoculated. This distribution sets the baseline for the overall effect of the different mutations in plant transcriptomes in absence of infection. The plant genotype showing the largest amount of genotype-specific DEGs was *dcl2 dcl3 dcl4* (2073), followed by *ddm1* (809), *polv* (462), and *jmj14* (258) (Supplemental Fig. S1B). Indeed, the number of genes altered in common by the three mutants affecting DNA methylation-related pathways was 313, 21.32% larger than the number of genes altered by the histone modification mutant. One-hundred sixty DEGs were in common for the four infected mutants. Remarkably, these genes were grouped into two large, well-balanced, clusters of over- and under-expressed genes highly consistent across the four mutant plants (Fig. 2 A) with only two genes presenting a different expression pattern between the mutants (left panel in Supplemental Fig. S1A).

Second, we sought to evaluate the differences with WT in the plants infected with the ancestral P1 viral populations (Fig. 2 B). The distribution of mutant-specific DEGs has significantly changed relative to the one observed for mock-inoculated plants (χ^2^ = 3828.937, 14 d.f., *P* < 0.001): now *ddm1* plants show the largest number of DEGs (1267), followed by *jmj14* (1143), *dcl2 dcl3 dcl4* (302), and *polv* (121). The subset of DEGs in common for the three infected DNA methylation-related mutants was now 27, which represents 97.64% less than the number of DEGs affected by the histone modification infected *jmj14* mutant. A total of 58 DEGs were shared by all four mutant plants after infection. These DEGs group in two major clusters, with an excess of overexpressed genes, although *ddm1* plants showed a group of eight underexpressed DEGs that were overexpressed in all the other mutants (Fig. 2 B, intermediate panel in Supplemental Fig. S1A).

Third, we compared the number of DEGs in plants infected with the evolved lineages (Fig. 2 C). In this case, the relevant comparison is between the number of DEGs observed for each plant genotype and ancestral and evolved viruses. Significant differences have been generated during the course of evolution (χ^2^ = 733.467, 14 d.f., *P* < 0.001), mostly driven by an increase in the relative number of *ddm1* (percentage deviation 4.9%) and *polv* (20.8%) specific DEGs along with a reduction in the relative numbers of *jmj14* (3.3%) and *dcl2 dcl3 dcl4* (26.7%). In this case, 74 DEGs were shared by all four infected mutant plant genotypes. These DEGs are clustered into two main categories: 17 over- and 57 under-expressed. However, the behavior of infected *jmj14* plants was remarkably different from the other mutants, with most of these DEGs being overexpressed (Fig. 2 C, right panel in Supplemental Fig. S1A). Overall, the percentage of DEGs shared by all mutants increased from the 1% in mock, to 21% in plants infected with the ancestral viruses and to 72% in plants inoculated with the evolved ones (Supplemental Fig. S1B), being the difference highly significant (χ^2^ = 109.360, 2 d.f., *P* < 0.001).

Finally, we evaluate the number of mutant-specific responses integrated with the degree of transcriptome perturbation, observing a differential pattern among mutants (Supplemental Fig. S1B). The direction of change, however, differed among genotypes: (*i*) the level of perturbation is higher in mock conditions than in infection for *dcl2 dcl3 dcl4* and *polv*, the most permissive genotypes [23] but not in the rest. (*ii*) The percentage of mutant-specific DEGs was higher in *ddm1* and *jmj14* mutants during infection (above 50%). And (*iii*) the percentage of mutant-specific DEGs seems to stabilize in *ddm1* (from 46% of specificity in mock conditions to 64% and 58% in evolved viral lineages) while continues growing in *jmj14* (from 24% in mock to 53% in ancestral and 68% in evolved viruses). In agreement with the observation that TuMV rate of evolution was faster in *jmj14* [23], these results confirm the stronger selective pressure that this mutant imposes on the virus.

For the sake of simplicity, hereafter we will focus on a reduced core of DEGs shared by all plant genotypes: six among all three treatments, five between infected plants but not with mock-inoculated plants and two that are shared between mock-inoculated and ancestral-inoculated plants (Fig. 2 D-E).

### Epigenetic mutants exhibit changes in a reduced set of common genes

Figure 2 D illustrates the overlapping among the 13 core DEGs. The six overexpressed DEGs shared by all plant genotypes, regardless of their infection status, are shown in Fig. 2 E(a). These six loci (Supplemental Table S1) can be seen as nonspecific responses of plants, regardless of their epigenetic context, to the inoculation process and growth conditions. This list includes *METHIONINE OVERACCUMULATION 1 (MTO1) RESPONDING DOWN 1* (*MRD1*), a gene that encodes for a protein involved in down-regulation of methionine biosynthesis, and is essential for salicylic acid (SA)-mediated defense [49], a ribosomal protein L34e superfamily member (AT3G28899), the ubiquitin family protein gene *1-PHOSPHATIDYLINOSITOL 4-KINASE C3* (*PI4KC3*), involved in regulation of flower development and responses to abscisic acid [50] and three noncoding RNAs (AT2G01422, AT4G04221 and AT4G04223). Interestingly, *MRD1*, *PI4KC3*, AT2G01422, AT3G28899, and AT4G04223 have previously been shown to be directly regulated by RdDM [46, 51, 52].

Figure 2 E(b) shows the expression pattern of the five core DEGs shared only by infected plants irrespective of virus’ evolution status (Supplemental Table S1). These five DEGs would represent shared responses to infection. This list includes genes related to stresses (AT1G22990, AT3G09450 and AT4G15390), meiosis (AT1G53490) and the methyltransferase *MET2*. AT1G53490, AT4G15390 and *MET2* are consistently induced upon infection with both the ancestral and evolved viruses in all plant genotypes. Changes of expression direction due to virus evolution is observed for the gene AT1G22990 in *ddm1*, *jmj14* and *polv*, indicating a potential role in the adaptation process in these particular genotypes. The same holds true for the gene AT3G09450 in the DNA methylation-related *dcl2 dcl3 dcl4* and *polv* mutants. Among the five genes, one (AT1G53490) is predicted to be directly regulated by POLV [46].

Two core DEGs are shared by mock- and ancestral-inoculated plants, but not with the evolved-inoculated ones (Fig. 2 D; Supplemental Table S1). Therefore, these two DEGs can be seen as either targets or drivers of TuMV adaptation to specific mutant plant genotypes. AT1G75945 and AT2G33175 both encode for mitochondrial membrane-associated hypothetical proteins that both were overexpressed compared to WT plants, except in *dcl2 dcl3 dcl4* plants, in which the first one was underexpressed (Fig. 2 E(c)). Notably, the ~ 4-fold increase in expression observed in *polv* plants agrees with the previous observation by Corrêa et al. [46].

### Impact of viral adaptation into plant responses

**Fig. 3:**
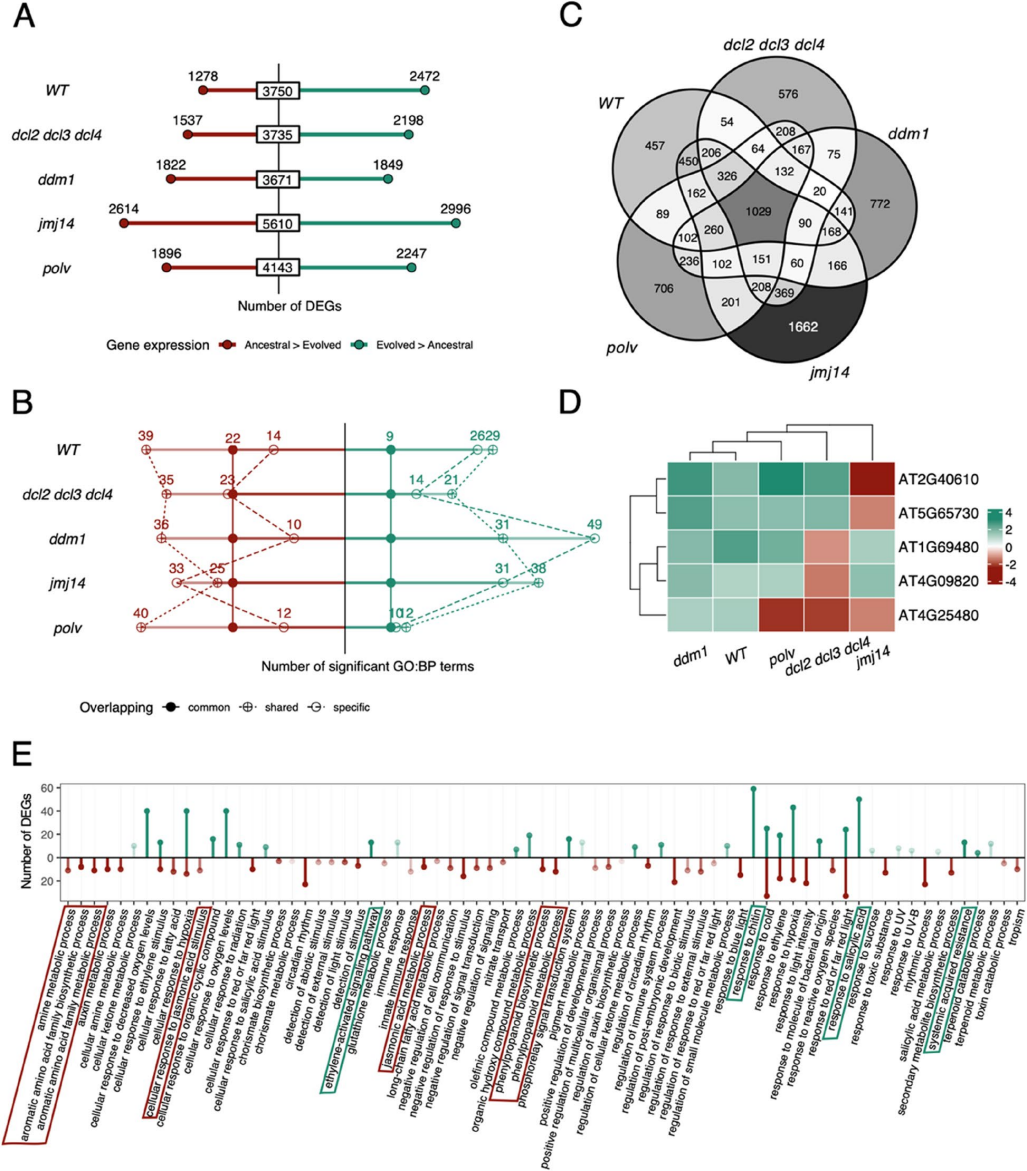
Characterization of DEGs between plants infected with the ancestral and evolved viruses, per plant genotype. **A** Number of repressed (red) and activated (green) DEGs elicited by viral adaptation in each plant genotype. **B** Number of GO biological processes terms within the repressed (red) and activated (green) DEGs enriched per plant genotype, indicating the number of common to all plant genotypes (solid symbols), shared by more than one plant genotype (crossed symbols), and specific of a single plant genotype (open symbols). **C** Distribution of DEGs across plant genotypes. **D** Only five of the 1029 DEGs common to all genotypes, present an opposite gene expression. The color scale represents the level of expression in log_2_*FC* as in (A) and (B). **E** GO biological processes terms from DEGS in common indicated in panel (C). In green, enriched categories within the activated DEGs, in red enriched categories within the repressed DEGs (adjusted *P* < 0.05

Once the differences between mutants and WT plants in their response to infection where explored, we focused on the expression changes elicited by the adaptation of the viral lineages to their corresponding local host genotypes [Fig. 1 purple lines: infected with evolved viral lineages (P12) vs. infected with their corresponding ancestors (P1)]. In this context, by up-regulated we meant significantly more expressed in plants infected with the evolved lineages with respect to plants infected with their corresponding ancestors; by down-regulated we meant the opposite. To simplify wording, we will refer to these genes as activated or repressed by the viral adaptation. The total number of virus-adaptation DEGs was larger in *jmj14* and *polv* than in WT (Fig. 3 A) while, at the other extreme, *dcl2 dcl3 dcl4* and *ddm1* showed a similar degree of disruption than WT plants (Fig. 3 A). However, it is noticeable that in all four mutants, the distributions of differentially activated and repressed genes were different than observed in WT plants (Fisher’s exact tests, *P* < 0.001). Overall, in all mutant plant genotypes, the number of repressed genes was larger than in WT plants. The situation was more variable for activated genes, with *dcl2 dcl3 dcl4*, *ddm1* and *polv* showing less than WT plants but *jmj14* showing more.

A functional enrichment analysis (GO: biological process) of the DEGs separated by its regulation revealed some categories common to all the genotypes (Supplemental Fig. S2). In the one hand, SAR, response to SA, response to hypoxia, or response to chitin were activated in the evolved viruses, while amine metabolic process, cellular response to hypoxia and to cold, and cellular response to jasmonic acid stimulus were enriched in the group of DEGs repressed in the evolved viruses. In the other hand, some categories were found only in specific genotypes, e.g., cutin biosynthetic process, isoprenoid and tetrapyrrole metabolic processes were enriched within the DEGs activated by the viral adaptation in WT but not in the mutants. *ddm1* presented the largest number of specific terms within the DEGs activated in evolved lineages (e.g., carotenoid related processes, cellular homeostasis or ketone biosynthetic processes) while *jmj14* was the genotype with more specific terms within the DEGs repressed (e.g., cell wall biogenesis, sulfur compound biosynthetic process or tropism between the specifically enriched terms) (Fig. 3 B, Supplemental Fig. S2).

### A core of genes associated with differences in TuMV adaptation was found differentially expressed in all plant genotypes

From a total of 9409 DEGs, we found a set of 1029 genes whose expression was significantly affected by the degree of TuMV adaptation, independently of the plant genotype in which evolution was carried on (Fig. 3 C). This core of genes showed a consistent profile of up- (48.0%) and down-regulation (51.5%) across all plant genotypes, except for five genes (Fig. 3 D). These exceptions were (Fig. 3 D, Table S2): AT1G69480 and AT4G09820 both specifically repressed in *dcl2 dcl3 dcl4* but activated in all other genotypes, AT2G40610 and AT5G65730 only repressed in *jmj14* but activated in the rest of genotypes, and AT4G25480 repressed in *dcl2 dcl3 dcl4*, *jmj14* and *polv* but activated in WT and *ddm1* plants.

The GO biological processes associated with the set of genes that increase their expression with virus adaptation in all genotypes include response to chitin, response to SA, SAR, response to ethylene signaling pathway and phosphorelay signal transduction system (Fig. 3 E and Supplemental Fig. S2). Among the shared genes whose expression has been repressed by viral adaptation, processes related with amine and aromatic amino acid family biosynthesis, jasmonic acid metabolic process, auxin metabolic processes, circadian rhythm, response to blue light, or response to toxic substances were observed (Fig. 3 E and Supplemental Fig. S2). Categories such as cellular response to hypoxia, cellular response to ethylene stimulus, response to cold, or response to red or far-red light appeared enriched both in activated and repressed genes (Fig. 3 E and Supplemental Fig. S2).

### Classification of genes according to their profile of gene expression in non-infected plantsvs. plants infected with the ancestral or the evolved viruses

**Fig. 4:**
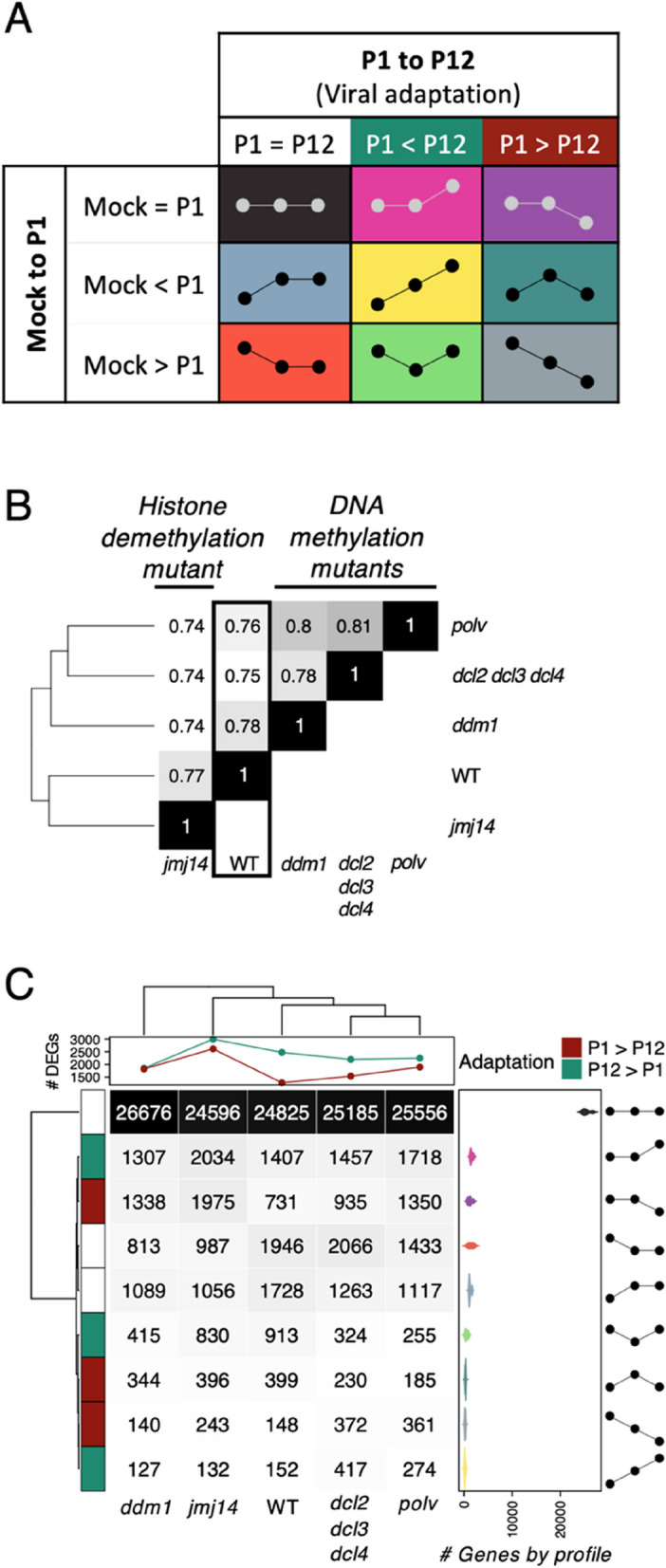
Classification of gene expression profiles according to the nine possible patterns of variation across conditions. **A** Classification profiles based on the direction of changes between the three inoculation conditions, mock-inoculated, ancestral (P1) virus-infected and evolved (P12) viruses-infected. **B** Hierarchical clustering of plant genotypes based on the similarity amongst their classification profiles in panel (A). **C** Abundance of each profile per plant genotype

In order to get a wider picture of the effect of infection with viral lineages at different degrees of adaptation to their local host genotypes, we considered the DEGs across the three inoculation conditions (Fig. 1 green lines) using the following approach. According to the inoculum type, i.e., mock, ancestral and evolved viruses, genes can be classified into nine basic profiles based on the direction of the changes between each pair of conditions, as illustrated in Fig. 4 A. Figure 4 B shows the clustering of the five plant genotypes according to the similarity between the genes classified into each of the nine profiles. DNA methylation mutants *dcl2 dcl3 dcl4*, *ddm1* and *polv* cluster together (*polv* share 81% with *dcl2 dcl3 dcl4* and an 80% with *ddm1*) while the histone demethylation mutant *jmj14* shows a more dissimilar distribution of profiles.

Figure 4 C shows all DEGs found on each of the plant genotypes classified into each of the nine possible expression patterns when comparing between mock-inoculated, ancestral- and evolved-infected plants. By far, in all five genotypes the most common pattern corresponded to genes that showed no differences between mock-inoculated and virus-infected plants, followed in WT by genes that responded to infection in the same magnitude (up or down) both for the ancestral and the evolved viruses. The most interesting cases are precisely those in which the pattern showed differences between WT and mutant plants inoculated with ancestral and evolved viruses. Fisher’s exact tests were used to evaluate pairwise differences between the patterns of DEGs shown for WT and each of the four mutant plant genotypes. In all four cases, the number of DEGs up or down found in the ancestral and the evolved inoculated mutant plants was different than observed for WT plants (Fisher’s exact test, *P* < 0.001 in all comparisons). Indeed, the number of up- and down-regulated DEGs due to virus evolution relative to the number of DEGs not associated to virus evolution (Fig. 4 C) is 2.75-fold largest for *jmj14* followed by 1.93-fold for *ddm1*, while the smallest difference was found for WT plants (1.02-fold). This observation further supports that TuMV adaptation to histone demethylation mutant plants resulted in a stronger genome-wide alteration in gene expression than adaptation to WT plants.

Next, we sought to functionally categorize the DEGs specific to each of the nine patterns in Fig. 4 C. Supplemental Fig. S3, present the results of the functional enrichment GO analyses per pattern for each plant genotype. For example, comparing across plant genotypes, a pervasively enriched GO term was photosynthesis processes though classified into different profiles: downregulated upon infection in *dcl2 dcl3 dcl4*, *polv* and WT, downregulated only by evolved infection in *jmj14* and upregulated in evolved-infected *ddm1* plants. Responses to chitin also showed an upregulation pattern for *ddm1* plants infected with the evolved viral lineages but not in the other genotypes. As an additional example of highly variable profiles, responses to SA signaling were always upregulated upon infection with evolved viruses, except in *ddm1* plants, and with *polv* plants showing upregulation also when infected with the ancestral viruses.

To delve deeper into functional responses potentially influenced by virus adaptation, we specifically examined each plant genotype and focused on differences between virus ancestral- and evolved-infected plants (Supplemental Fig. S4). In WT plants, only responses to cold were significantly altered by both types of viral lineages. Processes related to O_2_ levels and SA were overrepresented in evolved-infected plants, while processes related to membrane transport and organization, endocytosis, rRNA processing, and cytoplasmic translation were underrepresented in evolved-infected plants. In *dcl2 dcl3 dcl4*, responses to chitin, and hypoxia were specifically overrepresented in evolved-infected plants, while processes related to circadian rhythms and the transition from vegetative to reproductive phases in meristems were underrepresented in the same plants. This observation aligns with the impact of TuMV infection on the transition from vegetative growth to reproduction, resulting in plant castration [53]. *ddm1* plants exhibited the shortest list of significant GO terms, with only photosynthesis being shared by three of the expression profiles, two of which were overrepresented in evolved-infected plants compared to their ancestral counterparts. The histone modification-deficient *jmj14* plants displayed a diverse array of GO terms across the nine expression patterns. For example, responses to chitin, hypoxia, SA, jasmonic acid, and SAR were overrepresented in evolved-infected plants compared to ancestral-infected ones. In contrast, photosynthesis, response to cold, biogenesis of ribonucleoprotein complexes, rRNA processing and metabolism, and, notably, brassinosteroid-mediated signaling were underrepresented among evolved-infected plants. Finally, in *polv* plants, plastids organization and microtubule-related processes were specifically overrepresented in evolved-infected plants, while endomembrane system organization, Golgi vesicle transport, and processes related to the transition from vegetative to reproductive phases in meristems and regulation of multicellular differentiation were significantly underrepresented among evolved-infected plants compared to ancestral-infected ones. In short, an analysis of the intersections by profile shows that, avoiding the no-changes profile, the pattern with more genes in common among genotypes was represented by the repression in plants infected with ancestral viruses but not change with evolution (242 genes in common) while the less convergent was the upregulation through all conditions (6 genes in common) (Supplemental Fig. S5).

### DNA but not histone methylation affects the expression of genes nearby TEs

**Fig. 5:**
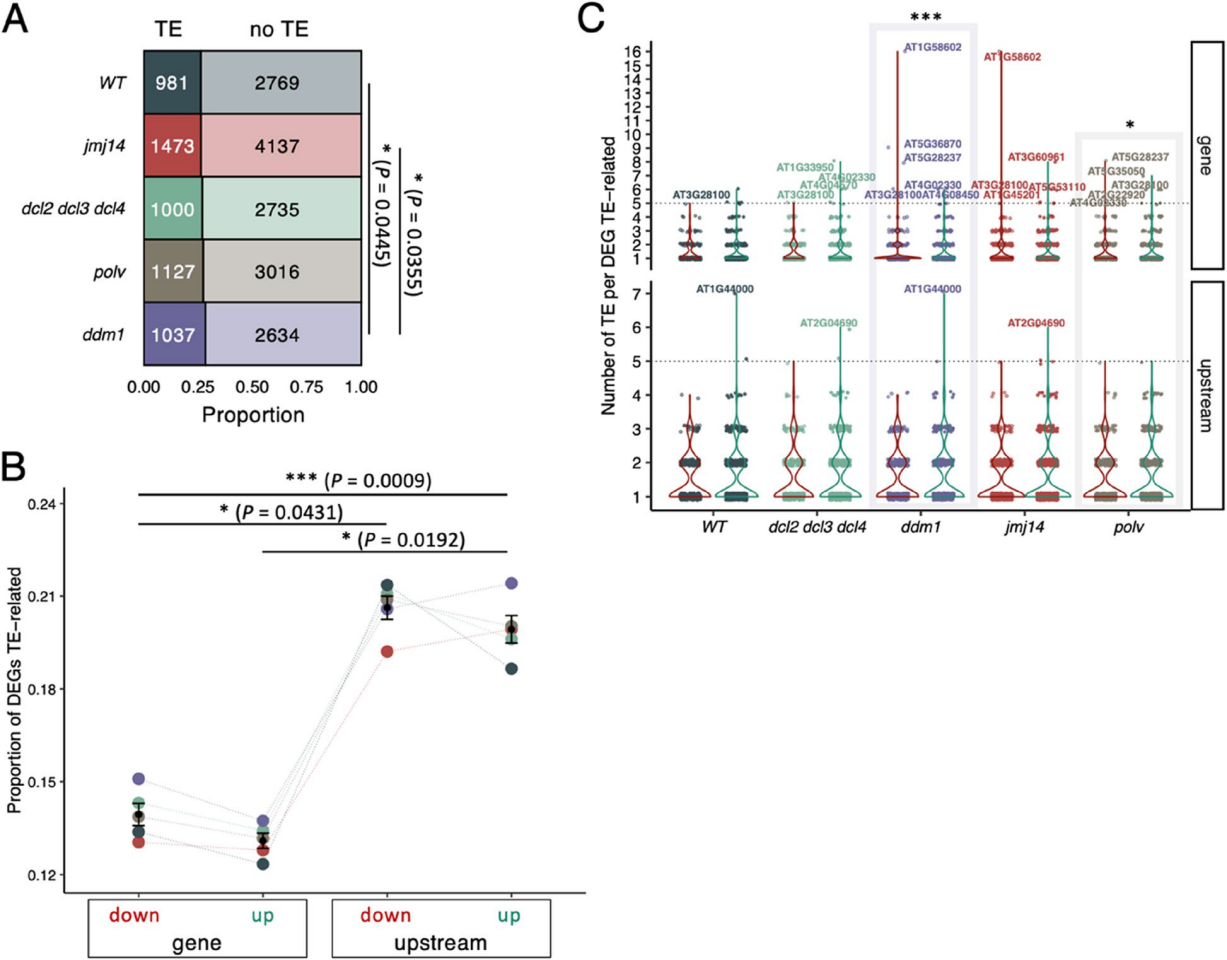
Analysis of DEGs (contrasting evolved vs. ancestral viruses) in the context of their association to TEs. **A** Proportion of DEGs in close proximity to TEs in each plant genotype. *ddm1* present a higher number of DEGs TE-associated than WT and *jmj14*. **B** Interaction between the position of TEs in genes (inside or within 1 Kbp upstream of their TSS) and the direction of regulation (upregulated or downregulated in the evolved lineages). Values are normalized by the total number of DEGs with the same locus and regulation. *P* values of Kruskall-Wallis rank sum test are indicated over the significant contrasts. **C** Distribution of DEGs related to the presence of TEs. Violin plots color represents DEGs-TE related within the activated (green) and repressed (red) ones. *ddm1* and *polv* showed significant differences in the distribution of its TEs depending on the localization and the expression where the TEs are associated (Kruskal-Wallis, *P* < 0.001 and *P* = 0.001, respectively)

In a prior study, Corrêa et al. [21] observed that in WT plants infected with evolved TuMV lineages, but not with their ancestral counterparts, TEs concentrated in centromeric and pericentromeric regions were induced early after infection. At later stages of infection, both induction and repression of different TE families were observed. Subsequently, Corrêa et al. [46] demonstrated that POLV target genes had a higher percentage of TE marks around their transcription start sites (TSS) than other genes. In contrast, genes regulated by JMJ14 showed a lower level of marks around their TSS. These observations suggest that genes found to be upregulated in infected DNA methylation mutants, especially in *ddm1*, but less in *jmj14* plants, are likely to be controlled by TE-related mechanisms. To further investigate this hypothesis, we sought differences in the number of DEGs with a TE within 1 Kbp upstream of their TSS between virus ancestral- and evolved-infected plants (Fig. 5 A). First, a highly significant correlation exists between the total number of DEGs and the number of DEGs in the proximity of TEs (Fig. 5 A: Pearson’s *r* = 0.990, 3 d.f., *P* = 0.001). The proportion of DEGs associated with TE was slightly higher in all the mutants than in WT plants, but this difference was significantly enriched only in *ddm1* plants (Fig. 5 A: Fisher’s exact test, *P* = 0.045). However, when comparing the TE-related DEGs between the three DNA methylation mutants and the histone modification mutant *jmj14*, also *ddm1* showed a significant augment in the number of TE-associated DEGs responding differentially to ancestral and evolved viral populations (Fig. 5 A: Fisher exact test, *P* = 0.036). The observed differences in the number of DEGs close to TEs in *ddm1* plants could be attributed to the higher basal instability of these genetic elements and the stronger transcriptome disturbance caused by the evolved virus (Supplemental Fig. S1B) in this genotype compared to the others.

Subsequently, we categorized the DEGs close to TEs based on their regulation (upregulated or downregulated in the evolved lineages) and the position of the TEs in the gene (whether they were located inside or within 1 Kbp upstream TSS) (Fig. 5 B). Significant differences exist between the number of DEGs containing a TE inside or upstream the gene (Kruskal-Wallis test: χ^2^ = 6.818, 1 d.f., *P* < 0.001), the sign of the regulation of the TE-related DEGs (Kruskal-Wallis test: χ^2^ = 4.811, 1 d.f., *P* = 0.028) and the interaction between both TE location and the sign of regulation (Kruskal-Wallis test: χ^2^ = 16.006, 3 d.f., *P* = 0.001). A Dunn *post hoc* pairwise comparisons test found significant differences between the location of TEs (in down-regulated, *P* = 0.043; in up-regulated, *P* = 0.019) but the regulation component only was significant between different locus (*P* < 0.001) (Fig. 5 B). No significant differences between genotypes were found in any group. The data suggest that TEs near gene promoters are more likely to correlate with changes in gene expression than those within genes.

**Fig. 6:**
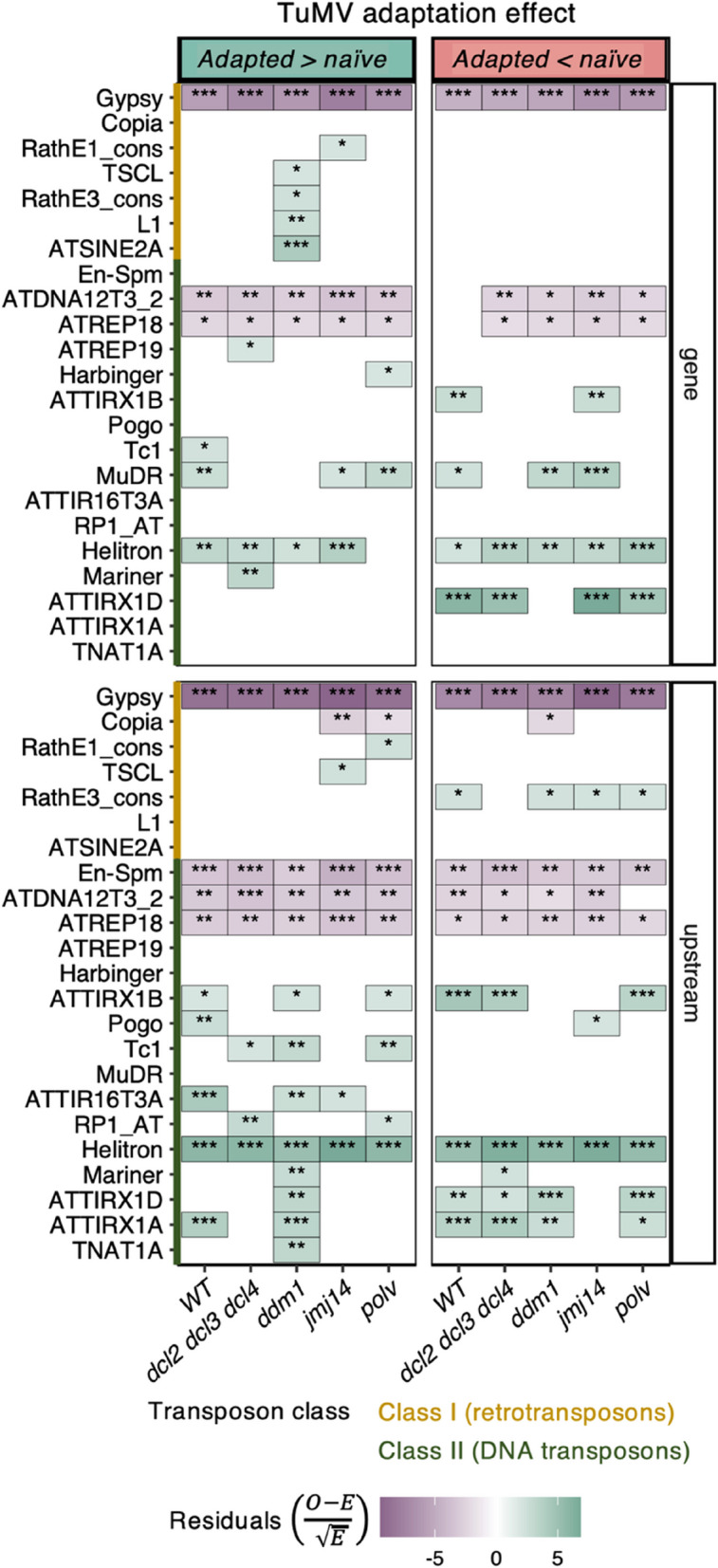
Analysis of the TE families linked DEGs associated to differences between ancestral and evolved viral isolates. Asterisks indicate significance levels in a χ^2^ goodness-of-fit test: ^*^P < 0.05, ^**^P < 0.01 and ^***^P < 0.001

Additionally, we checked the number of TEs associated with each TE-related DEG. This data revealed that although the proportion of DEGs having TEs in proximity of TSS is higher than the ones having TEs inside the genes (Fig. 5 B), the total counts of TEs in these same DEGs is higher inside than upstream the genes (Fig. 5 C: Wilcoxon rank test with continuity correction, *P* < 0.001). After separating by sign of regulation and localization, *ddm1* (Kruskal-Wallis, *P* < 0.001) and *polv* (Kruskal-Wallis, *P* = 0.001) presented differences within its four possible combinations (Fig. 5 C). The DEG with the highest density of TEs found inside its sequence (16 of both classes of TE) was *RECOGNITION OF PERONOSPORA PARASITICA 7* (*RPP7*), a gene that encodes an LRR and NB-ARC domains-containing disease resistance protein (Fig. 5 C) [54]. *RPP7* appeared as repressed by the evolved viruses adapted in *ddm1* and *jmj14* mutant plants. This gene is known to be regulated by a particularly interesting epigenetics mechanism in which a “domesticated” TE located in its first intron must be properly silenced in order to produce full-length transcripts [55]. Out of these 16 TEs inside *RPP7*, half belonged to the MuDR superfamily (Fig. 6) and more precisely to the VANDAL transposons. VANDAL transposons have coevolved mechanisms to escape the epigenetic silencing [56]. It is noteworthy that in *jmj14* and WT plants both activated and repressed genes were enriched in MuDR inside coding regions (Fig. 6). However, in *polv* plants only activated genes were enriched in MuDR while in *ddm1* plants, MuDR enrichment was only for repressed genes.

### Class II TEs are significantly enriched among Arabidopsis genes responding to TuMV adaptation while class I TEs were significantly deprived

An enrichment analysis of the types of TEs associated with the DEGs associated with virus adaptation revealed a general pattern of overrepresentation of class II elements (DNA transposons) and underrepresentation of class I ones (retrotransposons). A detailed analysis by superfamilies of TEs is shown in Fig. 6. The distribution of TE families across all the genome was used as the null model (expected values) and the proportion found at each condition (observed values) was tested with a χ^2^ goodness-of-fit. Briefly, from class I, Gypsy appears less than expected (*P* < 0.001), no matter the sign of regulation, localization relative to the gene coding sequence, or the plant genotype. An interesting exception is *ddm1* plants, where ATSNE2 (SINE type) and L1 (LINE type) were significantly enriched within the activated genes (Fig. 6: *P* < 0.001 and *P* = 0.008, respectively).

In contrast with this deprivation in class I TEs, a general enrichment in class II transposons was observed in all four plant genotypes, both upstream the TSS and inside the DEGs (*P* < 0.001). Members of the Helitron superfamily were the most abundantly over-represented type (Fig. 6). Helitrons (alike ssDNA viruses, bacteria and plasmids) use rolling circle replication for its propagation. This mechanism, in contrast with the cut-and-paste strategy of other DNA transposons, allows the retention of the original TE while the daughter TEs is copied. This mechanism allows TEs to capture gene fragments that can derive in epigenetic conflict [57, 58]. Cases of epigenetic conflict in the stress response context driven by Helitrons have been previously reported. For example, ATREP2 TEs have been found enriched in genes regulating ISR responses to herbivores [57, 59].

A GO enrichment analysis of the biological processes of these TE-proximal DEGs (Supplemental Fig. S7A) showed that the response to SA appeared overrepresented in all five plant genotypes. The same occurs with various categories related to hypoxia and the response to chitin, represented in genes related to both classes of TE. Responses to ethylene and to cold were also overrepresented in at least four of the five plant genotypes (Supplemental Fig. S7A). *ddm1* plants presented the largest number of terms enriched within the DEG TE-related elicited by the viral adaptation and none within the repressed ones (Supplemental Fig. S7B). Its more specific categories are related to photosynthesis. In contrast, *polv* present the largest number of enriched terms within the TE-related DEGs repressed in the evolved viruses (Supplemental Fig. S7B). In this case, genes are related to class I TE and all the functional categories are related to metabolic processes (Supplemental Fig. S7A).

### Putative alteration of the TuMV-*A. thaliana* protein-protein interaction network due to virus adaptation

The way in which virus and host interact changes with the degree of virus’ adaptation, which indeed depends on the fine tuning in the interactions between viral and host factors [27–29]. In order to explore the extent to which the target genes of TuMV adaptation could be perturbing the virus-host interaction network, we integrated the transcriptomic results described above with the protein-protein interaction (PPI) data in [43].

**Fig. 7:**
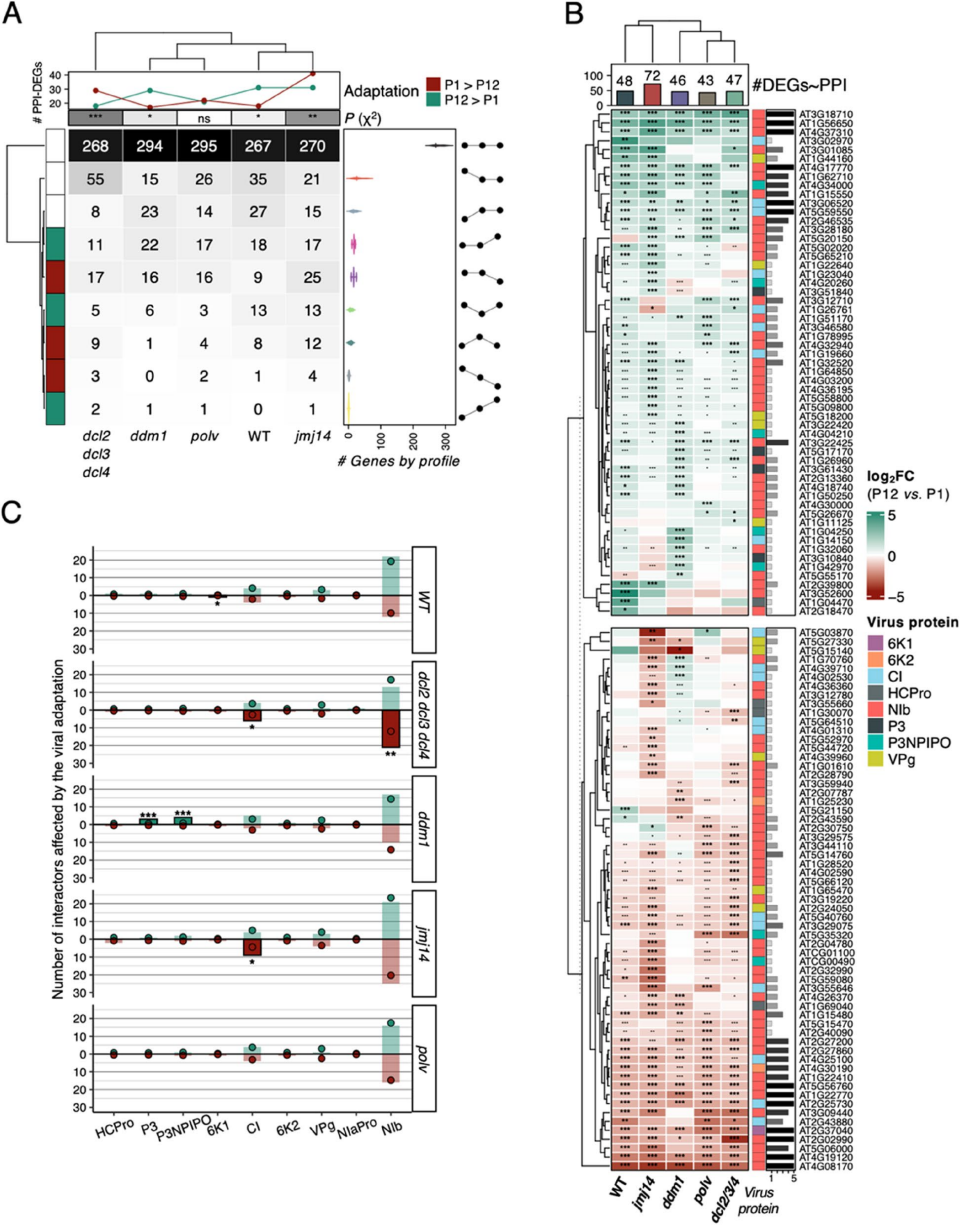
Interactors in the host-virus PPI network are altered by the adaptation of the virus. **A** Distribution of the PPI-related genes in the nine profiles classification developed in this work. The expression profiles (Fig. 4 A) of the genes involved in the PPI, were found to significantly differ from the genome-wide expression in four host genotypes, except in the case of *polv* mutant [χ^2^ homogeneity tests; *dcl2 dcl3 dcl4* (*P* < 0.001), *ddm1* (*P* = 0.011), *jmj14* (*P* = 0.002), *polv* (*P* = 0.271) and WT (*P* = 0.045)]. **B** Gene expression of DEGs PPI-associated. Asterisks indicate significant changes (adjusted *P* < 0.05), when |log_2_*FC*| > 1, bolded. The lateral bars represent the number of genotypes with the interactor significantly affected (adjusted *P* < 0.05 and |log_2_*FC*| > 1). **C** Observed (bars) and expected (points) host interactors for each viral protein were tested to search for significant differences using a χ^2^ homogeneity test. Asterisks indicate significance level: ^*^P < 0.05, ^**^P < 0.01 and ^***^P < 0.001. P1: ancestral viruses, P12: evolved viruses

Figure 4 A depicts expression profiles for DEGs whose proteins have been demonstrated to interact with viral proteins. The nine derived profiles differ significantly from the genome-wide expression in four host genotypes, except for the *polv* mutant [χ^2^ homogeneity tests; *dcl2 dcl3 dcl4* (*P* < 0.001), *ddm1* (*P* = 0.011), *jmj14* (*P* = 0.002), *polv* (*P* = 0.271), and WT (*P* = 0.045)] (Fig. 7 A). Focusing on the DEGs showing differences between virus ancestral- and evolved-infected plants, we found in infected *ddm1* and WT plants more PPI-associated DEGs activated within the viral adaptation, while *dcl2 dcl3 dcl4* and *jmj14* were enriched in more repressed genes (Fig. 7 B). We used the number of genes activated and repressed by the evolved viral lineages as a proxy of the probabilities for gene activation and repression in each host genotype. With these probabilities, we calculated the expected number of interactors altered for each viral protein (Fig. 7 C) and used a χ^2^ homogeneity test to evaluate whether some of the viral proteins were enriched or depleted in selected DEGs. P3 and P3N-PIPO showed more interactions than expected with proteins encoded by the overexpressed genes in *ddm1* (χ^2^ homogeneity tests, *P* < 0.001 for both proteins), an over-representation of repressed genes was found for 6K1 in WT (*P* = 0.031), for CI and NIb in *dcl2 dcl3 dcl4* (*P* = 0.029 and *P* = 0.007, respectively), as well as for CI in *jmj14* (*P* = 0.021). Despite this, *ddm1* is the only genotype with significantly more upregulated interactors than expected by share chance.

Finally, Supplemental Fig. S8, maps the effects of viral adaptation in the host transcriptome over the host-virus PPI network. Our previous work showed that TuMV VPg is a target of virus adaptation to different genotypes of *A. thaliana*, to its susceptibility to infection or to environmental conditions through an increase in its fitness and/or virulence [22, 23, 53, 60]. In the present PPI network analysis, five repressed and four activated interactors were found for VPg, although none of them was shared by all plant genotypes. It is noteworthy that four out of the five repressed interactors displayed binding functions, highlighting the *FAS1* subunit of the chromatin assembly factor 1 (*CAF-1*), that has a remarkable histone chaperone function whose defect induced an altered plant epigenetic landscape [61], and the *eIFIso(4G2)* translation initiation factor. It is well known that potyviruses require translation initiation proteins of the eIF4F/eIF4E/eIF4G complex including the scaffolding eIFIso(4G2) protein. Recently, it has been showed a connection between the levels/degradation of some of these translation initiation proteins and the increase or restriction of TuMV accumulation in *A. thaliana* infected plants [62]. Regarding the VPg induced interactors it highlights a HSP40/DNAJ chaperone which plays a role in viral pathogenicity.

By contrast, it should be noted that thirteen altered interactors were found in the five plant genotypes (Supplemental Table S3). Viral replicase NIb had five repressed (with diverse functions, but remarkably a methyltransferase superfamily member) and four activated interactors (including transcription factor MYB75 and E3 ubiquitin ligase), multifunctional CI two activated (E3 ubiquitin ligase and an Agenet domain-containing protein involved in chromatin remodeling) and one repressed (Zinc finger FYVE domain containing protein), and 6K1 a repressed one, *PHENYLALANINE AMMONIA-LYASE 1* (*PAL1*). A further exploration of these extensive results would be beyond the scope of this article and the focus of future work, but we refer readers to the information shown in Supplemental Table S2. However, in this complex context we wanted to highlight the significant virus-host interaction involving 6K1 and PAL1. It is relevant to note that 6K1 is the only viral protein for which only one interactor has been found, shared by all genotypes, for which an unexpected biological function has been reported recently [63]. Pentamers of 6K1 had been demonstrated to be a functional viroporin [63]. Viroporins are small hydrophobic proteins that insert in membranes modifying its permeability and are essential for viral pathogenicity, affecting inflammasome formation, inducing apoptosis and autophagy, and evading immune responses [64]. Their role in viral pathogenicity and inflammatory responses has been assessed in several viruses [65], as well as their potential as drug targets [66]. 6K1, contrary to the majority of the known viroporins, also plays an essential role in the viral replication cycle [67]. 6K1 interactor PAL1 is a phenylalanine ammonia-lyase that acts upstream of the phenylpropanoid pathway. This pathway was found to be consistently repressed in plants infected with the evolved TuMV populations (Fig. 3 and Supplemental Fig. S3), thus suggesting its importance in viral adaptation. Among other diverse roles, this pathway has been shown to be redundant in synthetizing SA and to be involved in resistance to fungal [68] and viral [69] pathogens. Concretely, the overexpression of *PAL1* in cassava increases resistance to cassava brown streak virus, another member of the *Potyviridae* family [69]. Despite the character of the interaction 6K1 - PAL1 remains unknown, the repression of *PAL1* expression by the evolved lineages in the five plant genotypes suggests *PAL1* as a promising candidate for future studies.

### Concluding remarks

Evolution profoundly alters how plant viruses interact with their hosts [21, 27–29, 60]. Building on earlier studies [23], our comprehensive analysis of large transcriptomic datasets reveals that changes in epigenetic regulatory pathways play a crucial role in shaping the evolution of RNA viruses. We identified a core set of genes that influence TuMV adaptation in *A. thaliana*, independent of the specific genetic makeup of the plant’s epigenetic pathways. Additionally, we found many genes whose impact on viral adaptation varies depending on the host genotype. Our results highlight the significant role of the JMJ14 histone demethylase in driving TuMV evolution. Furthermore, our analysis uncovered a strong association between genes regulated by epigenetic marks and the presence of TEs located within or near genes involved in TuMV adaptation, particularly evident in *ddm1* mutant plants. By integrating transcriptomic data with PPIs in the same pathosystem, we identified additional host genes critical for TuMV adaptation. Notably, our study emphasizes the importance of the interaction between viroporin 6K1 and PAL1 in facilitating virus adaptation.

## Supplementary Information

Supplementary Material 1.

## Data Availability

Raw Illumina RNA-Seq data generated for this study are available in NCBI SRA under BioProject accession PRJNA974369.

## References

[1] Soosaar JLM, Burch-Smith TM, Dinesh-Kumar SP. Mechanisms of plant resistance to viruses. Nat Rev Microbiol. 2005;3:789–99. 16132037 10.1038/nrmicro1239

[2] Zhou JM, Zhang Y. Plant immunity: danger perception and signaling. Cell. 2020;181:978–89. 32442407 10.1016/j.cell.2020.04.028

[3] Carr JP, Lewsey MG, Palukaitis P. Signaling in induced resistance. Adv Virus Res. 2010;76:57–121. 20965072 10.1016/S0065-3527(10)76003-6

[4] Loebenstein G. Local lesions and induced resistance. Adv Virus Res. 2009;75:73–117. 20109664 10.1016/S0065-3527(09)07503-4

[5] Kachroo P, Chandra-Shekara AC, Klessig DF. Plant signal transduction and defense against viral pathogens. Adv Virus Res. 2006;66:161–91. 16877061 10.1016/S0065-3527(06)66004-1

[6] López-Gomollon S, Baulcombe DC. Roles of RNA silencing in viral and non-viral plant immunity and in the crosstalk between disease resistance systems. Nat Rev Mol Cell Biol. 2022;23:645–62. 35710830 10.1038/s41580-022-00496-5

[7] Voinnet O. RNA silencing as a plant immune system against viruses. Trends Genet. 2001;17:449–59. 11485817 10.1016/s0168-9525(01)02367-8

[8] García-Ruiz H, Takeda A, Chapman EJ, Sullivan CM, Fahlgren N, Brempelis KJ, Carrington JC. Arabidopsis RNA-dependent RNA polymerase and dicer-like proteins in antiviral defense and small interfering RNA biogenesis during *turnip mosaic virus* infection. Plant J. 2010;22:481–96. 10.1105/tpc.109.073056 PMC2845422 20190077

[9] Borges F, Martienssen RA. The expanding world of small RNAs in plants. Nat Rev Mol Cell Biol. 2015;16:727–41. 26530390 10.1038/nrm4085 PMC4948178

[10] Hung YH, Slotkin RK. The initiation of RNA interference (RNAi) in plants. Curr Opin Plant Biol. 2021;61:102014. 33657510 10.1016/j.pbi.2021.102014

[11] Liu P, Cuerda-Gil D, Shahid S, Slotkin RK. The epigenetic control of the transposable element life cycle in plant genomes and beyond. Annu Rev Genet. 2022;56:63–87. 36449356 10.1146/annurev-genet-072920-015534

[12] Böhmdorfer G, Sethuraman S, Rowley MJ, Krzyszton M, Rothi MH, Bouzit L, Wierzbicki AT. Long non-coding RNA produced by RNA polymerase V determines boundaries of heterochromatin. eLife. 2016;5: e19092. 27779094 10.7554/eLife.19092 PMC5079748

[13] Cuerda-Gil D, Slotkin RK. Non-canonical RNA-directed DNA methylation. Nat Plants. 2016;2:16163. 27808230 10.1038/nplants.2016.163

[14] Bond DM, Baulcombe DC. Small RNAs and heriTable epigenetic variation in plants. Trends Cell Biol. 2014;24:100–7. 24012194 10.1016/j.tcb.2013.08.001

[15] Gong Z, Morales-Ruiz T, Ariza RR, Roldán-Arjona T, David L, Zhu JK. *ROS1*, a repressor of transcriptional gene silencing in Arabidopsis, encodes a DNA glycosylase/lyase. Cell. 2002;111:803–14. 12526807 10.1016/s0092-8674(02)01133-9

[16] Saze H, Shiraihi A, Miura A, Kakutani T. Control of genic DNA methylation by a jmjC domain containing protein in *Arabidopsis thaliana*. Science. 2008;319:462–5. 18218897 10.1126/science.1150987

[17] Lu F, Cui X, Zhang S, Liu C, Cao X. JMJ14 is an H3K4 demethylase regulating flowering time in Arabidopsis. Cell Res. 2010;20:387–90. 20177424 10.1038/cr.2010.27

[18] Lloyd JPB, Lister R. Epigenome plasticity in plants. Nat Rev Genet. 2021;23:55–68. 34526697 10.1038/s41576-021-00407-y

[19] Diezma-Navas L, Pérez-González A, Artaza H, Alonso L, Caro E, Llave C, Ruiz-Ferrer V. Crosstalk between epigenetic silencing and infection by tobacco rattle virus in Arabidopsis. Mol Plant Pathol. 2019;20:1439–52. 31274236 10.1111/mpp.12850 PMC6792132

[20] Leone M, Zavallo D, Venturuzzi A, Asurmendi S. RdDM pathway components differentially modulate *Tobamovirus* symptom development. Plant Mol Biol. 2020;104:467–81. 32813230 10.1007/s11103-020-01051-6

[21] Corrêa RL, Sanz-Carbonell A, Kogej Z, Müller SY, Ambrós S, López-Gomollón S, Gómez G, Baulcombe DC, Elena SF. Viral fitness determines the magnitude of transcriptomic and epigenomic reprograming of defense responses in plants. Mol Biol Evol. 2020;37:1866–81. 32259238 10.1093/molbev/msaa091

[22] Navarro R, Ambrós S, Butković A, Carrasco JL, González R, Martínez F, Wu B, Elena SF. Defects in plant immunity modulate the rates and patterns of RNA virus evolution. Virus Evol. 2022;8:veac059. 35821716 10.1093/ve/veac059 PMC9272744

[23] Ambrós S, Olmo-Uceda MJ, Corrêa RL, Elena SF. Phenotypic and genomic changes during *turnip mosaic virus* adaptation to *Arabidopsis thaliana* mutants lacking epigenetic regulatory factors. Evolution. 2024;78:69–85. 37891007 10.1093/evolut/qpad192

[24] Ivanov KI, Eskelin K, Lõhmus A, Mäkinen K. Molecular and cellular mechanisms underlying potyvirus infection. J Gen Virol. 2014;95:1415–29. 24722679 10.1099/vir.0.064220-0

[25] Revers F, García JA. Molecular biology of potyviruses. Adv Virus Res. 2015;92:101–99. 25701887 10.1016/bs.aivir.2014.11.006

[26] Pagán I, Fraile A, Fernández-Fueyo E, Montes N, Alonso-Blanco C, García-Arenal F. *Arabidopsis thaliana* as a model for the study of plant-virus co-evolution. Philos Trans R Soc B. 2010;365:1983–95. 10.1098/rstb.2010.0062 PMC2880114 20478893

[27] Agudelo-Romero P, Carbonell P, Pérez-Amador MA, Elena SF. Virus adaptation by manipulation of host’s gene expression. PLoS ONE. 2008;3:e2397. 18545680 10.1371/journal.pone.0002397 PMC2398778

[28] Cervera H, Ambrós S, Bernet GP, Rodrigo G, Elena SF. Viral fitness correlates with the magnitude and direction of the perturbation induced in the host’s transcriptome: the tobacco etch potyvirus-tobacco case study. Mol Biol Evol. 2018;35:1599–615. 29562354 10.1093/molbev/msy038 PMC5995217

[29] Hillung J, García-García F, Dopazo J, Cuevas JM, Elena SF. The transcriptomics o fan experimentally evolved plant-virus interaction. Sci Rep. 2016;6: 24901. 27113435 10.1038/srep24901 PMC4845063

[30] Chen CC, Chao CH, Chen CC, Ye SD, Tsai HT, Chang CA. Identification of turnip mosaic virus isolates causing yellow stripe and spot on calla lily. Plant Dis. 2003;87:901–5. 30812792 10.1094/PDIS.2003.87.8.901

[31] Boyes DC, Zayed AM, Ascenzi R, McCaskill MJ, Hoffman NE, Davis KR, Görlach J. Growth stage-based phenotypic analysis of Arabidopsis: a model for high throughput functional genomics in plants. Plant Cell. 2001;13:1499–510. 11449047 10.1105/TPC.010011 PMC139543

[32] Ewels P, Magnusson M, Lundin S, Käller M. MultiQC: summarize analysis results for multiple tools and samples in a single report. Bioinformatics. 2016;32:3047–8. 27312411 10.1093/bioinformatics/btw354 PMC5039924

[33] Li H. Aligning sequence reads, clone sequences and assembly contigs with BWA-MEM. arXiv 2013, 1303.3997v2. 10.48550/arXiv.1303.3997.

[34] Danecek P, Bonfield JK, Liddle J, Marshall J, Ohan V, Pollard MO, Whitwham A, Keane T, McCarthy SA, Davies RM, Li H. Twelve years of SAMtools and BCFtools. Gigascience. 2021;10: giab008. 33590861 10.1093/gigascience/giab008 PMC7931819

[35] McKenna A, Hanna M, Banks E, Sivachenko A, Cibulskis K, Kernytsky A, Garimella K, Altshuler D, Gabriel S, Daly M, DePristo MA. The genome analysis toolkit: a MapReduce framework for analyzing next-generation DNA sequencing data. Genome Res. 2010;20:1297–303. 20644199 10.1101/gr.107524.110 PMC2928508

[36] Dobin A, Davis CA, Schlesinger F, Drenkow J, Zaleski C, Jha S, Batut P, Chaisson M, Gingeras TR. STAR: Ultrafast universal RNA-seq aligner. Bioinformatics. 2013;29:15–21. 23104886 10.1093/bioinformatics/bts635 PMC3530905

[37] Love MI, Huber W, Anders S. Moderated estimation of old change and dispersion for RNA-seq data with DESeq2. Genome Biol. 2014;15:550. 25516281 10.1186/s13059-014-0550-8 PMC4302049

[38] Coenye T. Do results obtained with RNA-sequencing require independent verification? Biofilm. 2021;3: 100043. 33665610 10.1016/j.bioflm.2021.100043 PMC7823214

[39] Everaert C, Luypaert M, Maag JLV, Cheng QX, Dinger ME, Hellemans J, Mestdagh P. Benchmarking of RNA-sequencing analysis workflows using whole-transcriptome RT-qPCR expression data. Sci Rep. 2017;7:1559. 28484260 10.1038/s41598-017-01617-3 PMC5431503

[40] Szklarczyk D, Gable AL, Nastou KC, Lyon D, Kirsch R, Pyysalo S, Doncheva NT, Legeay M, Fang T, Bork P, Jensen LJ, von Mering C. The STRING database in 2021: customizable protein-protein networks, and functional characterization of user-uploaded gene/measurements sets. Nucleic Acids Res. 2021;49:D605-12. 33237311 10.1093/nar/gkaa1074 PMC7779004

[41] Wu T, Hu E, Xu S, Chen M, Guo P, Dai Z, Feng T, Zhou L, Tang W, Zhan L, Fu X, Liu S, Bo X, Yu G. clusterProfiler 4.0: a universal enrichment tool for interpreting omics data. Innovation. 2021;2:100141. 34557778 10.1016/j.xinn.2021.100141 PMC8454663

[42] Lawrence M, Huber W, Pagès H, Aboyoun P, Carlson M, Gentleman R, Morgan MT, Carey VJ. Software for computing and annotating genomic ranges. PLoS Comput Biol. 2013;9: e1003118. 23950696 10.1371/journal.pcbi.1003118 PMC3738458

[43] Martínez F, Carrasco JL, Toft C, Hillung J, Giménez-Santamarina S, Yenush L, Rodrigo G, Elena SF. A binary interaction map between turnip mosaic virus and *Arabidopsis thaliana* proteomes. Commun Biol. 2023;6:28. 36631662 10.1038/s42003-023-04427-8 PMC9834402

[44] Shannon P, Markiel A, Ozier O, Baliga NS, Wang JT, Ramage D, Amin N, Schwikowski B, Ideker T. Cytoscape: a software environment for integrated models of biomolecular interaction networks. Genome Res. 2003;13:2498–504. 14597658 10.1101/gr.1239303 PMC403769

[45] Zemach A, Ki MY, Hsieh PH, Coleman-Derr D, Eshed-Williams L, Thao K, Harmer SL, Zilberman D. The Arabidopsis nucleosome remodeler DDM1 allows DNA methylatransferases to access H1-containing heterochromatin. Cell. 2013;153:193–205. 23540698 10.1016/j.cell.2013.02.033 PMC4035305

[46] Corrêa RL, Kutnjak D, Ambrós S, Bustos M, Elen SF. Identification of epigenetically regulated genes involved in plant-virus interaction and their role in virus-triggered induced resistance. BMC Plant Biol. 2024;24:172. 38443837 10.1186/s12870-024-04866-3 PMC10913459

[47] Stroud H, Greenberg MVC, Feng S, Bernatavichute YV, Jacobsen SE. Comprehensive analysis of silencing mutants reveals complex regulation of the Arabidopsis methylome. Cell. 2013;152:352–64. 23313553 10.1016/j.cell.2012.10.054 PMC3597350

[48] Greenberg MVC, Deleris A, Hale CJ, Liu A, Feng S, Jacobsen SE. Interplay between active chromatin marks and RNA-directed DNA methylation in *Arabidopsis thaliana*. PLoS Genet. 2013;9: e1003946. 24244201 10.1371/journal.pgen.1003946 PMC3820799

[49] Singh A, Sharma A, Singh N, Nandi AK. MTO1-RESPONDING DOWN 1 (MRD1) is a transcriptional target of OZF1 for promoting salicylic acid-mediated defense in Arabidopsis. Plant Cell Rep. 2022;41:1319–28. 35325291 10.1007/s00299-022-02861-2

[50] Akhter S, Uddin MN, Jeong IS, Kim DW, Liu XM, Bahk JD. Role of AtPI4Kgamma3, a type II phosphoinositide 4-kinase, in abiotic stress responses and floral transition. Plant Biotechnol J. 2015;14:215–30. 25879253 10.1111/pbi.12376 PMC11389056

[51] Blevins T, Pontvianne F, Cocklin R, Podicheti R, Chandrasekhara C, Yerneni S, Braun C, Lee B, Rusch D, Mockaitis K, Tang H, Pikaard CS. A two-step process for epigenetic inheritance in Arabidopsis. Mol Cell. 2014;54:30–42. 24657166 10.1016/j.molcel.2014.02.019 PMC3988221

[52] Kurihara Y, Matsui A, Kawashima M, Kaminuma E, Ishida J, Morosawa T, Mochizuki Y, Kobayashi N, Toyoda T, Shinozaki K, Seki M. Identification of the candidate genes regulated by RNA-directed DNA methylation in Arabidopsis. Biochem Biophys Res Commun. 2008;376:553–7. 18805399 10.1016/j.bbrc.2008.09.046

[53] Melero I, González R, Elena SF. Host developmental stages shape the evolution of a plant RNA virus. Philos Trans R Soc B. 2023;378:20220005. 10.1098/rstb.2022.0005 PMC9979778 36744567

[54] Liu J, Kim BM, Kaneko Y, Inukai T, Masuta C. Identification of the TuNI gene causing systemic necrosis in Arabidopsis ecotype L*er* infected with *turnip mosaic virus* and characterization of its expression. J Gen Plant Pathol. 2015;81:180–91.

[55] Tsuchiya T, Eulgem T. An alternative polyadenylation mechanism coopted to the Arabidopsis *RPP7* gene through intron retrotransposon domestication. Proc Natl Acad Sci USA. 2013;110:E3535-43. 23940361 10.1073/pnas.1312545110 PMC3773791

[56] Sasaki T, Ro K, Caillieux E, Manabe R, Bohl-Viallefond G, Baduel P, Colot V, Kakutani T, Quadrana L. Fast co-evolution of anti-silencing systems shapes the invasiveness of Mu-like DNA transposons in eudicots. EMBO J. 2022;41:e110070. 35285528 10.15252/embj.2021110070 PMC9016345

[57] Barro-Trastoy D, Köhler C. Helitrons: genomic parasites that generate developmental novelties. Trends Genet. 2024;40:437–48. 38429198 10.1016/j.tig.2024.02.002

[58] Coates BS. Horizontal transfer of a non-autonomous helitron among insect and viral genomes. BMC Genomics. 2015;16:137. 25766741 10.1186/s12864-015-1318-6 PMC4344730

[59] Wilkinson SW, Hannan Parker A, Muench A, Wilson RS, Hooshmand K, Henderson M, Moffat EK, Rocha PSCF, Hipperson H, Stassen JHM, López Sánchez A, Fomsgaard IS, Krokene P, Mageroy MH, Ton J. Long-lasting memory of jasmonic acid-dependent immunity requires DNA demethylation and ARGONAUTE1. Nat Plants. 2023;9:81–95. 36604579 10.1038/s41477-022-01313-9

[60] Carrasco JL, Ambrós S, Gutiérrez PA, Elena SF. Adaptation of turnip mosaic virus to *Arabidopsis thaliana* involves rewiring of VPg-host proteome interactions. Virus Evol. 2024;10:veae055. 39091990 10.1093/ve/veae055 PMC11291303

[61] Franek M, Nešpor Dadejová M, Pírek P, Kryštofová K, Dobisová T, Zdráhal Z, Dvořáčková M, Lochmonová G. Histone chaperone deficiency in Arabidopsis plants triggers adaptive epigenetic changes in histone variants and modifications. Mol Cell Proteom. 2024;23:100795. 10.1016/j.mcpro.2024.100795 PMC11263794 38848995

[62] Zafirov D, Giovinazzo N, Lecampion C, Field B, Ducassou JN, Couté Y, Browning KS, Robaglia C, Gallois JL. Arabidopsis eIF4E1 protects the translational machinery during TuMV infection and restricts virus accumulation. PLoS Pathog. 2023;20:e1011417. 10.1371/journal.ppat.1011417 PMC10721207 37983287

[63] Chai M, Li L, Li Y, Yang Y, Wang Y, Jiang X, Luan Y, Li F, Cui H, Wang A, Xiang W, Wu X, Cheng X. The 6-kilodalton peptide 1 in plant viruses of the family *Potyviridae* is a viroporin. Proc Natl Acad Sci USA. 2024;121: e2401748121. 38739789 10.1073/pnas.2401748121 PMC11127057

[64] Xia X, Cheng Z, Wang M, Ou X, Sun D, Mao S, Huang J, Yan Q, Wu Y, Chen S, Zhang S, Zhu D, Jia R, Liu M, Zhao XX, Gao Q, Tian B. Functions of viroporins in the viral life cycle and their regulation of host cell responses. Front Immunol. 2022;13:890549. 35720341 10.3389/fimmu.2022.890549 PMC9202500

[65] Nieva JL, Madan V, Carrasco L, Viroporins. Structure and biological functions. Nat Rev Microbiol. 2012;10:563–74. 22751485 10.1038/nrmicro2820 PMC7097105

[66] To J, Surya W, Torres J. Targeting the channel activity of viroporins. Adv Prot Chem Struct Biol. 2016;104:307–55. 10.1016/bs.apcsb.2015.12.003 PMC7102763 27038378

[67] Cui H, Wang A. Plum pox virus 6K1 protein is required for viral replication and targets the viral replication complex at the early stage of infection. J Virol. 2016;90:5119–31. 26962227 10.1128/JVI.00024-16 PMC4859702

[68] Ferrari S, Plotnikova JM, de Lorenzo G, Ausubel FM. Arabidopsis local resistance to Botrytis cinerea involves salicylic acid and camalexin and requires EDS4 and PAD2, but not SID2, EDS5 or PAD4. Plant J. 2003;35:193–205. 12848825 10.1046/j.1365-313x.2003.01794.x

[69] Kavil S, Otti G, Bouvaine S, Armitage A, Maruthi MN. *PAL1* gene of the phenylpropanoid pathway increases resistance to the *Cassava brown streak virus* in cassava. Virol J. 2021;18:184. 34503522 10.1186/s12985-021-01649-2 PMC8428094

